# Integrating mental health services into primary health care – a review of challenges and outcomes in the international setting

**DOI:** 10.1192/j.eurpsy.2021.1076

**Published:** 2021-08-13

**Authors:** L. Moreno, A. Sousa

**Affiliations:** 1 Department Of Psychiatry And Mental Health, Setúbal Hospital Center, Setúbal, Portugal; 2 Castelo Family Healthcare Unit (usf Castelo), ACeS Arrábida, Sesimbra, Portugal

**Keywords:** Mental Health Services, Healthcare organization, primary health care, Mental Health integration

## Abstract

**Introduction:**

Mental illness accounts for about one-third of the world’s disability, a burden that many health systems cannot adequately respond to. Up to 70% of mental health (MH) patients are followed-up in primary health care (PHC) settings. To bridge the treatment gap, the World Health Organization developed mhGAP, a guidance package for integrated management of priority MH disorders in lower-income countries. Other countries have developed their own evidence-based interventions.

**Objectives:**

Overviewing countries’ strategies towards integrating MH services into PHC, their outcomes and challenges.

**Methods:**

Review of literature using PubMed search terms “mental health primary care”, MeSH terms “Primary Health Care”, “Mental Health Care” and “organization and administration”, published in the last 5 years, in English.

**Results:**

25 of 602 articles were selected. The mhGAP programme has seen successful integration in pilot district-level programs, but wider implementation has stalled due to stigma and lack of clinical engagement, resources, MH specialists, and policy support. The Quebec MH reform promoted integrated service networks, improving accessibility and quality of care (QoC). A Norwegian-Russian long-standing collaboration initiative has significantly improved treatment for anxiety and depression (A&D), with 58% reliable recovery rate. A Danish collaborative care intervention provided high-quality treatment of moderate A&D. In Peru, a similar initiative allowed early detection, referral, and treatment of MH patients attending PHC services.

**Conclusions:**

Comprehensive, integrated and responsive collaborative care models are a cost-efficient strategy to improve QoC for many MH conditions across diverse populations. MH-PHC integration initiatives have seen varying degrees of success. However, several barriers impact wider implementation and scale-up.

